# Appropriateness of prescribed oral antibiotic duration at the time of hospital discharge

**DOI:** 10.1017/ash.2022.245

**Published:** 2022-06-29

**Authors:** Carly E. Sedlock, Marissa J. Cavaretta, Alexander J. Haines, Kevin B. Nguyen, Neelesh Agarwal, Jason C. Gallagher

**Affiliations:** 1 Infectious Disease Associates, St. Luke’s University Health Network, Bethlehem, Pennsylvania; 2 School of Pharmacy, Temple University, Philadelphia, Pennsylvania

## Abstract

Antibiotic stewardship initiatives usually occur in the inpatient setting and should be optimized during transitions of care. In this study, we assessed the appropriateness of oral antibiotic treatment duration at the time of discharge from our institution based on national guidelines and clinical parameters for common infections.

Antimicrobial stewardship programs have been implemented to combat the growing threat of antimicrobial resistance.^1,2^ However, stewardship initiatives usually occur in the inpatient setting and are often lacking at the time of transitions of care (ie, at hospital discharge).^3^ Studies have demonstrated that up to two-thirds of treatment durations for common infections are completed after patients are discharged from the hospital and that a significant number of these antibiotic prescriptions are inappropriate in terms of duration of therapy or choice of agent.^3,4^ This inappropriate antibiotic prescribing leads to increased adverse events, antimicrobial resistance, and costs.^1^ In this study, we assessed the appropriateness of antibiotic treatment duration at the time of patient discharge from our institution based on published guidelines and clinical parameters for common infections.

## Methods

This retrospective chart review included 300 adult patients discharged from Temple University Hospital (a 700-bed academic tertiary-care center in Philadelphia, Pennsylvania) who were on oral antibiotics for acute infections from January to March 2019. Patients aged 18 years or older with a hospital stay ≥48 hours were included in this study. Included patients had a documented discharge diagnosis of one of the following infections: community-acquired pneumonia (CAP), hospital-acquired pneumonia (HAP), cystitis, pyelonephritis, skin and soft-tissue infection (SSTI), intra-abdominal infection (IAI), chronic obstructive pulmonary disease (COPD) exacerbation, bronchitis, or pharyngitis.

Patients were excluded who met the following criteria: admission to the emergency department or intensive care unit only; hospital length of stay >30 days (to exclude complicated cases); a diagnosis of cystic fibrosis, pregnancy, human immunodeficiency virus (HIV) with CD4 <200, or receipt of chronic immunosuppression (organ transplant recipient, splenectomy, neutropenia, receiving >10 mg prednisone or equivalent for >30 days); diagnosis of endocarditis, osteomyelitis, or prosthetic joint infection; bloodstream infection caused by *Staphylococcus aureus* or any organism from an unclear source; antibiotics provided for long-term suppression or prophylaxis; discharge on IV antibiotics or discharge against medical advice; or treatment for a fungal pathogen.

The primary outcome evaluated in this study was total prescribed duration of antibiotic therapy, which was compared to the following variables: the duration recommended by clinical guidelines; the minimum supported by guidelines and clinical trials^5^; and the duration beyond the point of clinical stability, defined as normal vital signs with improvement in symptoms present from diagnosis. Clinical practice guidelines published by the Infectious Diseases Society of America (IDSA) were used to establish appropriate durations.^6–10^ Each indication and antibiotic was assessed using standards appropriate for the combination by a physician trained in infectious diseases. The study was approved by the Temple University Institutional Review Board.

## Results

In total, 300 eligible patient charts were evaluated; patients received 342 individual antibiotic prescriptions on discharge (Table 1). Of these 300 patients, 171 (57%) were women, and the median age was 59 years (interquartile range [IQR], 48–68). Moreover, 73% of inpatients received IV therapy and 76% received oral antibiotic therapy. Also, 70% of patients who were prescribed oral antibiotics were transitioned to these agents prior to hospital discharge. The most common antibiotics prescribed were fluoroquinolones, which accounted for 101 prescriptions (30%), followed by amoxicillin–clavulanate (n = 60, 18%), and azithromycin (n = 60, 18%).

**Table 1.** Patient Characteristics

Characteristic	Value
Total patients, no.	300
**Sex, no. (%)**	
Female	171 (57)
Male	129 (43)
Age, median y (IQR)	59 (48–68)
Charlson comorbidity index, median (IQR)	3.5 (1–6)
Duration of hospitalization, median d (IQR)	4 (2–5)
**Route of inpatient antibiotic administration, no. patients (%)**	
IV	218 (73)
Oral	228 (76)
Oral antibiotic on discharge was given in the hospital first, no. (%)	211 (70)
**Oral antibiotic prescriptions at discharge, no.**	342
Amoxicillin/clavulanate	60
Azithromycin	60
Levofloxacin	60
Ciprofloxacin	41
Doxycycline	40
Trimethoprim-sulfamethoxazole	26
Clindamycin	18
Cephalexin	13
Metronidazole	11
Cefpodoxime	5
Nitrofurantoin	3
Amoxicillin	2
Cefdinir	2
Cefuroxime	1

Note. IQR, interquartile range.

Community-acquired pneumonia (n = 67) and SSTI (n = 79) were the most common diagnoses for which antibiotics were prescribed, followed by COPD (n = 58), cystitis (n = 33), pyelonephritis (n = 30), IAI (n = 15), HAP (n = 9), and other (bronchitis, pharyngitis; n = 8) (Table 2). Of the 33 patients diagnosed with cystitis, 9 (27%) were determined to have asymptomatic bacteriuria based on clinical documentation and laboratory analysis.

**Table 2.** Antibiotic Durations

Days of Therapy, Median (IQR)									
Variable	Total (n=300)	SSTI (n=80)	CAP (n=67)	COPD (n=58)	Cystitis (n=33)	Pyelonephritis (n=30)	IAI (n=15)	HAP (n=9)	Other (n=8)^a^
Total antibiotic duration	8 (6–11)	10 (8–14)	7 (7–9)	5 (5–7)	7 (5–7)	13 (10–14)	10 (8.5–17)	7 (7–8)	9 (7.8–13.3)
Duration of oral outpatient antibiotics	5 (3–7)	7 (5–10)	5 (3.5–5)	3 (2–4)	4 (2–5)	7.5 (5–10)	7 (5–9.5)	3.5 (3–5)	6 (4.5–7.8)
Excess duration compared to guidelines	2 (0–4)	3 (1–6)	2 (2–3.5)	0 (0–0)	3 (0–5)	1 (0–4)	3 (1.5–10)	0 (0–1)	7.5 (5–8)
Excess duration compared to minimum possible	3 (1–5)	4 (2–6)	2 (2–4)	0 (0–1.8)	4 (2–6)	4 (1.3–5.8)	7 (5–13.5)	0 (0–1)	7.5 (5–8)
Duration received after point of clinical stability	6 (4–8)	8 (6–10)	6 (4.5–7)	4 (3–5)	5.5 (4–7)	9.5 (8–11.8)	8 (6.5–12.5)	5 (4–5)	7.5 (5.5–13)

Note. IQR, interquartile range; SSTI, skin and soft-tissue infection; CAP, community-acquired pneumonia; COPD, chronic obstructive pulmonary disease; IAI, intra-abdominal infection; HAP, hospital-acquired pneumonia.

a Bronchitis, upper-respiratory tract infection, pharyngitis.

Overall, patients received a median of 8 total days (IQR, 6–11) of antibiotics, including 5 days (IQR, 3–7) of oral antibiotics (Table 2). Patients received a median of 2 days (IQR, 0–4) of excess antibiotics compared to recommended IDSA guidelines, 3 days (IQR, 1–5) compared to the minimum possible duration based on clinical guidelines and published literature, and 6 days (IQR, 4–8) past the point of clinical stability. The greatest variability in prescribing was seen with SSTI and IAI, and the most inappropriate prescription durations were seen with SSTI, IAI, and cystitis.

## Discussion

Stewardship interventions enhance patient care through improved antimicrobial prescribing, ensuring the “right drug, right time, right dose, right duration.” In this retrospective chart review of 300 patients at a large tertiary-care center, antibiotics were given longer than necessary on hospital discharge for multiple acute conditions. This finding supports the results from several other studies; despite the growth of data supporting shorter courses of therapy for multiple common infections and the inclusion in clinical guidelines, patients are often prescribed longer courses than clinically indicated.^2,4,5^ This situation fosters antimicrobial resistance and increases the risk of adverse events, such as *Clostridioides difficile* infection. Given that most patients will complete a significant proportion of their antibiotic regimens after discharge from the hospital following clinical improvement, the transition of care from the inpatient to outpatient setting offers a critical opportunity for stewardship interventions to ensure that discharge prescriptions are appropriate. A pilot stewardship program targeting transition of care has been initiated at our institution to address this problem.

In addition to evaluating duration of therapy compared to recommendations from the literature and clinical guidelines, we also determined the duration prescribed past the point of clinical stability. We recognize the difficulty of determining this from chart review alone, as well as the paucity of evidence supporting this parameter in clinical practice. Guidelines are used to provide standardization in practice; however, these are meant to serve as a framework, and shorter or longer courses of therapy may be appropriate based on individual patient circumstances. More research is needed to investigate the use of clinical parameters to determine individual treatment courses rather than relying on fixed durations of therapy for every patient. Choice of agent was not a main outcome evaluated in this study; however, the high frequency of fluroquinolone prescriptions warrants further evaluation. Prescriptions for asymptomatic bacteriuria are also problematic and should be an area of intervention.

This study had several limitations. It was retrospective in nature and we determined clinical stability using chart review alone. Although we cannot determine the exact reasoning behind the choice of a specific duration, several studies have shown that antibiotic prescribing practices are often driven by learned preferences and habits rather than evidence.^5^ We also observed that some patients were discharged with an intended duration of therapy appropriate per guidelines, but the practitioner did not include inpatient days of therapy in the calculation of total duration. This is an issue that changes to the electronic medical record can help mitigate. Behavior and practice patterns are more difficult to change; however, education through direct face to face interaction with stewardship team members can play a role.

In summary, our study shows that patients are often prescribed antibiotics longer than necessary based on published guidelines, evidence from literature, and clinical parameters. Enhanced stewardship interventions are needed at critical periods such as transitions of care to improve antimicrobial prescribing and patient care.
